# Geraniol reverses obesity by improving conversion of WAT to BAT in high fat diet induced obese rats by inhibiting HMGCoA reductase

**DOI:** 10.1038/s41387-023-00254-2

**Published:** 2023-12-05

**Authors:** Shushmita Chand, Alok Shiomurti Tripathi, Tabinda Hasan, Kavitha Ganesh, Mary Anne W. Cordero, Mohammad Yasir, Magdi E. A. Zaki, Pankaj Tripathi, Lucy Mohapatra, Rahul Kumar Maurya

**Affiliations:** 1 Amity Institute of Pharmacy, Amity University, Sector 125, Noida, Uttar Pradesh India; 2 Department of Pharmacology. Era College of Pharmacy, Era University, Lucknow, UP India; 3 College of medicine, Princess Nourah Bint Abdulrahman University, Riyadh, Kingdom of Saudi Arabia; 4 Department of Chemistry, College of Science, Imam Mohammad lbn Saud Islamic University, Riyadh, Kingdom of Saudi Arabia; 5 Department of Pharmacology, Nootan Pharmacy College, Sankalchand Patel University, Visnagar, Gujarat 384315 India

**Keywords:** Obesity, Fat metabolism

## Abstract

**Objectives:**

Present report evaluates the protective effect of geraniol on high fat diet (HFD) induced obesity in rats and also determines the molecular mechanism of it.

**Methods:**

Rats were induced with obesity with administration of HFD for four weeks and geraniol 200 and 400 mg/kg p.o. was administered for the next four week in the respective groups. Blood glucose and oral glucose tolerance test (OGTT), lipid profile was estimated in the geraniol treated HFD induced obesity in rats. Moreover, docking study was performed to determine the specific mechanism of geraniol by targeting HMG-CoE A reductase (in silico).

**Results:**

There was significant increase in body weight and amelioration in altered serum glucose and lipid profile were observed in the geraniol treated group than negative control group. Weight of organs and adipose tissue isolated from different regions of the body was reduced in geraniol treated group than negative control. Moreover, geraniol interact with HMG-CoA reductase having binding energy −5.13.

**Conclusions:**

In conclusion, data of the report reveals that geraniol reduces obesity by promoting the conversion of white adipose tissue (WAT) to brown adipose tissue (BAT), as it interacts with HMG-CoA reductase in HFD induced obesity in rats.

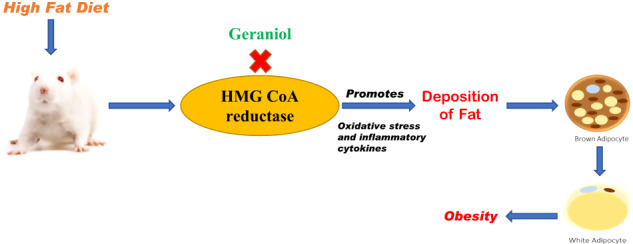

## Introduction

Obesity is an intricate multifactorial disease that results due to accumulation of fat causes negative effect on health. A report monitored for 22 years on 12,543 participants, suggest the increase in the prevalence of age-adjusted obesity up to 13.99% [1]. Metabolic disorders are one of the major risk factors associated with obesity [2]. Moreover, patients with obesity are at major risk for developing a range of diseases, including cardiovascular disease (CVD), gastrointestinal disorders, diabetes specifically Type 2 diabetes (T2DM) as well as several other metabolic related disorders [3]. When energy intake consistently outpaces energy expenditure (EE), obesity develops, with the excess energy deposited largely as triglycerides in white adipose tissue (WAT) [4]. There are several mechanisms involved in the conversion of WAT, as changes in the quantity and character of adipose tissue macrophages (ATMs) are linked to the onset of diabetes and obesity [5]. Recent studies have revealed that ATMs emit proinflammatory cytokines that are comparable to those produced by traditionally activated M1 macrophages, directly contributing to insulin resistance in Type 2 diabetes [6]. Moreover, M2 macrophages activation promotes thermogenesis in BAT, which contributes to the disruption of energy and lipolysis, promotes insulin resistance, and reduces obesity [7].

Hydroxymethylglutaryl-coenzyme A (HMG-CoA) reductase is an enzyme involved in the biosynthesis of cholesterol and inhibition of it with statins reduces synthesis of cholesterol, which also promotes sensitivity of insulin [8]. HMG-CoA reductase inhibition also reported to activates M2 level in the BAT. It also ameliorates obesity and promotes insulin sensitivity [9]. However chronic consumption of statins has several limitations, that necessitates the development of new therapy for the management of obesity and diabetes. Phytochemicals are known to be used for the management of chronic disorders. Geraniol is chemically an acyclic monoterpene (essential oil) reported to regulate immunomodulatory and anti-inflammatory activity [10]. Geraniol reported for depressant and neuroprotective effect to modulate behavioral cognitive function [11]. Moreover, it also shows inhibitory effect against HMG-CoA reductase and hypoglycaemic effect against both type 1 and 2 diabetic rat model [12, 13]. Thus, present study aims at evaluating the effect of geraniol on obesity.

## Materials and methods

### Experimental

Thirty healthy male Albino Wistar rats (180–200 gm) were maintained under controlled conditions (23 ± 2 °C, 55 ± 5%: humidity, 12 h L/D cycle). Institutional Animal Ethics Committee of Amity University Lucknow Campus approved the protocol (AUUP/AIP/4.2/2021). All animals were categorised into five groups as Normal control, Negative control, Geraniol 200 and 400 mg/kg and Standard (Atorvastatin 5 mg/kg) group. Obesity was induced with the administration of HFD (31% fat, 12% protein, 46% carbohydrate; 516.5 Kcal/100 g of feed) for the duration of four weeks excluding the normal control group [14]. At the end of fourth week blood glucose was measured by glucose oxidase peroxidase (GOD-POD) method in all the groups. Geraniol treated groups received geraniol 200 and 400 mg/kg p.o. [15] and Standard group received atorvastatin (20 mg/kg BW p.o.) for the duration of four weeks from fifth to eighth week. Blood glucose was estimated in all the groups by GOD-POD method at the end of fourth and eighth week of protocol. Feed intake was recorded daily, and body weight was monitored every week during complete protocol. Rats were fasted overnight for the estimation of OGTT at the end of protocol.

### Collection and preparation of blood sample

Blood was withdrawn through the retro orbital for blood glucose measurement. Further prior the day of sacrifice, the rats were fasted overnight, and the blood was collected from the lateral tail vein by puncturing with needle for estimation of oral glucose tolerance test (OGTT). Serum samples were collected from blood using a centrifuge operated at 3000 rpm for 10 min and stored in the refrigerator at 4 °C prior to biochemical analysis (blood glucose and lipid profile).

### Isolation of adipose tissue

The rats were sacrificed by cervical dislocation after eight weeks and restrained on dissection tray placing the dorsal side of body upward. The skin of rat was held up near the neck region and an incision was made with the help of scissor. The upper layer of skin was then cut open with the help of scapula and forceps. A butterfly shaped region of fat was exposed which was isolated to obtain the interscapular WAT and BAT. The rat was then pinned on the dissection tray facing the ventral side upward. Upper layer of skin was held up and incision was again made on the abdominal region to cut open the upper layer of skin by scrapping the sub cutaneous layer to obtain the sub cutaneous fat. The epididymal, inguinal, mesenteric, triceps, intraperitoneal adipose tissues were located and isolated by scrapping or by cutting out properly.

### Isolation of liver, pancreas and heart

The abdomen was cut open to the thoracic region with the help of scissor and organs were kept apart with the help of forceps. Diaphragm was punctured with scissor and pulled it away from ribs. Heart was isolated by making an incision using fine scissors. Ribs were cut from both sides and lung was removed with fine scissors so that the heart was exposed. Heart was pinched downward using forceps and heart tissue was perfused with cool phosphate until no more blood is visible in the coronary arteries. Then after liver and pancreas were pulled out of abdominal cavity. Liver was separated from diaphragm by cutting the falciform and coronary ligament that attached the liver to diaphragm. All these tissues were excised and washed in cold phosphate buffer saline (PBS) pH 7.4. The tissues were then dried in folds of tissue paper and weighed.

### Assessment of oxidative stress parameters

Oxidative stress parameters like MDA and SOD level were estimated in the homogenate of liver tissue as per previously reported methods [16]. Briefly, 2.5 mL Methionine, 0.3 mL Riboflavin, 0.1 mL NBT, 0.1 mL liver homogenate were uniformly illuminated with incandescent light for 15 min and level of SOD was estimated by determining absorbance at 560 nm. Moreover, lipid peroxidation was estimated by determine level of MDA with thiobarbituric acid at 532 nm wavelength.

### Histopathological study

The pancreas, heart, and adipose tissue were isolated, flushed with ice-cold PBS, and fixed in 10% neutral buffered formalin while the heart was fixed in 4% formalin. Using a microtome, tissue was paraffin embedded and cut into pieces 5 mm thickness. The sections were placed on glass slides coated with poly L-lysine and stained using H&E staining with a Nikon Eclipse 80i microscope (Nikon, Kawasaki, Japan), the slides were examined after being photomicrographed in three randomly selected fields from each slide.

### In silico

PubChem compound database (https://pubchem.ncbi.nlm.nih.gov/) was used to take the structure of geraniol in SDF format, which was converted with OpenBabel in to PDB format, which was further stored as pdbqt file, used for docking study. Protein Data Bank (PDB) utilized to retrieve the 3D X-ray crystal structure protein having 1HWK PDB code. Docking study was performed with Docking Autodock 4.2 software, protein was cleared from pdb chain A with Discovery Studio Visualizer v20.1.0.19295 software by removing Hetatms. Later, autodock 4.2 software was utilized to open the protein file, kollman charges and computing of Gasteiger charges were added after the addition of polar-H atoms. Macromolecule file was stored as Pdbqt file after merged all the non polar-H atom with autodock tool, this file was used for further study.

Active centres of protein were estimated with Autodock 4.2 and geraniol file was used as ligand, globally optimized conformation was identified with Lamarckian genetic search algorithm (LGA). The other parameters for docking calculation used were population size, 150; mutation rate, 0.02; and cross-over rate of 0.8; 2.5 × 105 operations were executed for the generation of new docking trail. Grid box was decided on the target protein with *X*, *Y* and *Z* coordinates. Ligand, a grid spacing of 0.375 Å was used around the docking area and binding energy of geraniol with protein was analysed with Autodock 4.2, which was used for the estimation of hydrophobic interaction and H-bonds between target protein and ligand.

### Statistical analysis

Data are expressed as mean ± SEM (*n* = 6). One-way analysis of variance (ANOVA) followed by post-hoc Dunnett test was used to compare the various groups. Two-way repetitive measure ANOVA followed by post-hoc Bonferroni test was used to find the differences in MWM acquisition data. *p* < 0.05 was considered statistically significant.

## Results

### Geraniol effect on body weight and feed intake

**Fig. 1:**
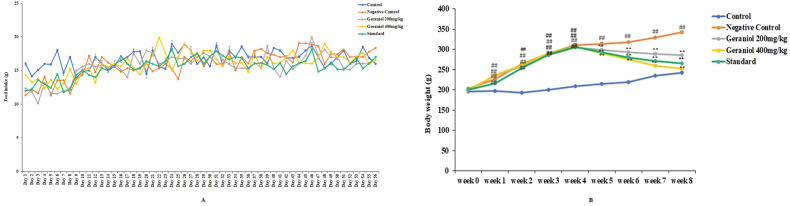
Assessment of geraniol effect on feed intake and body weight in HFD-induced obese rats. **A** Assessment of daily feed intake; **B** assessment of body weight every week. Mean ± SEM (*n* = 6); *p* < 0.01 Vs Control; **p < 0.01 vs negative control.

Feed intake and body weight was assessed in geraniol treated HFD induced obesity in rats as shown in Fig. 1A, B. Feed intake was monitored every day for the duration eight weeks, there was no specific significant (*p* < 0.01) alteration in feed intake observed among all the groups including control and HFD given groups (Fig. 1A). Body weight was reported to be enhanced significantly (*p* < 0.01) in negative control group than control group of rats, which was reversed in geraniol treated HFD induced obesity in rats (Fig. 1B).

### Geraniol effect on blood glucose

**Fig. 2:**
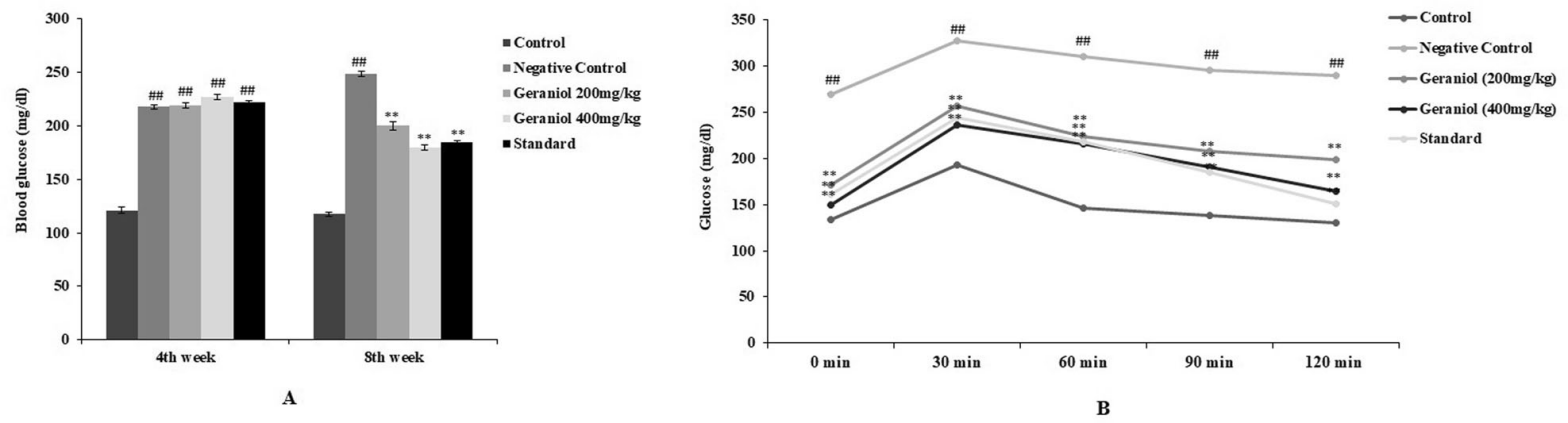
Assessment of geraniol effect on blood glucose and oral glucose tolerance test in HFD-induced obese rats. **A** Assessment of blood glucose at the end of 4th and 8th week of protocol; **B** assessment of glucose level by oral glucose tolerance test. Mean ± SEM (*n* = 6); *p* < 0.01 vs Control; *p* < 0.01 vs negative control.

Glucose level was estimated in the serum of geraniol treated HFD induced obesity in rats as shown in Fig. 2A, B. Estimation of glucose level was done at the end of 4th and 8th week of protocol in geraniol treated HFD induced obesity in rats to assess anti diabetic activity (Fig. 2A). Moreover, oral glucose tolerance test was performed at the end of protocol (Fig. 2B). There was significant (*p* < 0.01) increase in the level of glucose in all HFD exposed groups than control group at the end of 4th week, which was reversed in geraniol treated HFD induced obesity in rats. In OGTT, glucose level was estimated after 0, 30, 60, 90 and 120 min of administration of glucose in HFD induced obesity in rats, negative control group shows higher level of glucose than control group, which was reduced significantly (*p* < 0.01) in geraniol treated HFD induced obesity in rats.

### Geraniol effect on lipid profile

Lipid profile such as cholesterol, triglyceride, HDL and LDL was determined in the serum of geraniol treated HFD induced obesity in rats. Level of cholesterol (73.97 ± 1.34 mg/dl), triglyceride (116.18 ± 2.68 mg/dl), LDL (123.41 ± 4.46 mg/dl) was enhanced (*p* < 0.01) and reduction in HDL (20.20 ± 1.39 mg/dl) in the serum of negative control group than control group (Cholesterol: 32.98 ± 1.24 mg/kg; Triglyceride: 67.68 ± 0.66 mg/kg; HDL: 42.40 ± 2.69 mg/dl; LDL: 60.84 ± 2.69 mg/dl) of rats. Lipid profile (Cholesterol: 43.88 ± 1.65 mg/kg; Triglyceride: 81.01 ± 3.29 mg/kg; HDL: 36.20 ± 1.16 mg/dl; LDL: 71.36 ± 2.47 mg/dl) was attenuated in the serum of geraniol treated HFD induced obesity in rats (Table 1).

**Table 1:** Effect of geraniol on lipid profile in obese rats fed with HFD.

Sr. No.	Group	Cholesterol (mg/dl)	Triglycerides (mg/dl)	HDL-Cholesterol (mg/dl)	LDL-Cholesterol (mg/dl)
**1**	Control	32.98 ± 1.24	67.68 ± 0.66	42.40 ± 2.69	60.84 ± 2.69
**2**	Negative control	73.97 ± 1.34^##^	116.18 ± 2.68^##^	20.20 ± 1.39^##^	123.41 ± 4.46^##^
**3**	Geraniol (200 mg/kg)	65.05 ± 2.05^**^	99.86 ± 1.55^**^	24.60 ± 0.93^**^	99.67 ± 1.18^**^
**4**	Geraniol (400 mg/kg)	43.88 ± 1.65^**^	81.01 ± 3.29^**^	36.20 ± 1.16^**^	71.36 ± 2.47^**^
**5**	Standard	38.85 ± 1.23^**^	75.43 ± 3.45^**^	28.00 ± 0.89^**^	90.64 ± 1.65^**^

mean ± SEM (*n* = 6); ^##^*p* < 0.01 Vs Control; ^**^p < 0.01 Vs Negative control

### Geraniol effect on the weight of adipose tissue and organs

**Fig. 3:**
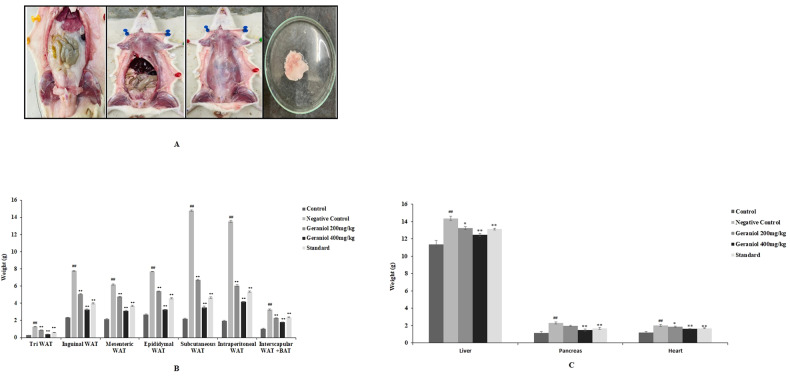
Assessment of geraniol effect on the weight of adipose tissue and organs like (liver, pancreas and heart) in HFD-induced obese rats. **A** Image of adipose tissue isolation; **B** Assessment of subcutaneous fat, the epididymal, inguinal, mesenteric, triceps, intraperitoneal adipose tissue weight; **C** Assessment of the weight of different organs liver, pancreas and heart. Mean ± SEM (*n* = 6); ^##^*p* < 0.01 Vs Control; **p < 0.01 vs negative control.

Adipose tissue from the different body regions like sub cutaneous fat, the epididymal, inguinal, mesenteric, triceps, intraperitoneal adipose tissues were isolated from rats of each group and weight was observed for them as shown in Fig. 3A, B. Negative control fed with HFD for eight weeks showed significant (*p* < 0.01) increase in weight of triceps, inguinal, subcutaneous, intraperitoneal, and interscapular fat mass compared to control group. Treatment with geraniol and standard for last four weeks resulted in significant (*p* < 0.01) decrease in isolated fat depots compared to negative control group.

Moreover, effect of geraniol was observed on the weight of organs like liver, pancreas and heart of HFD obesity in rats (Fig. 3C). There was significant (*p* < 0.01) increase in the weight of liver, pancreas and heart isolated from negative control group than control group of rats.

### Geraniol effect on markers of oxidative stress

**Fig. 4:**
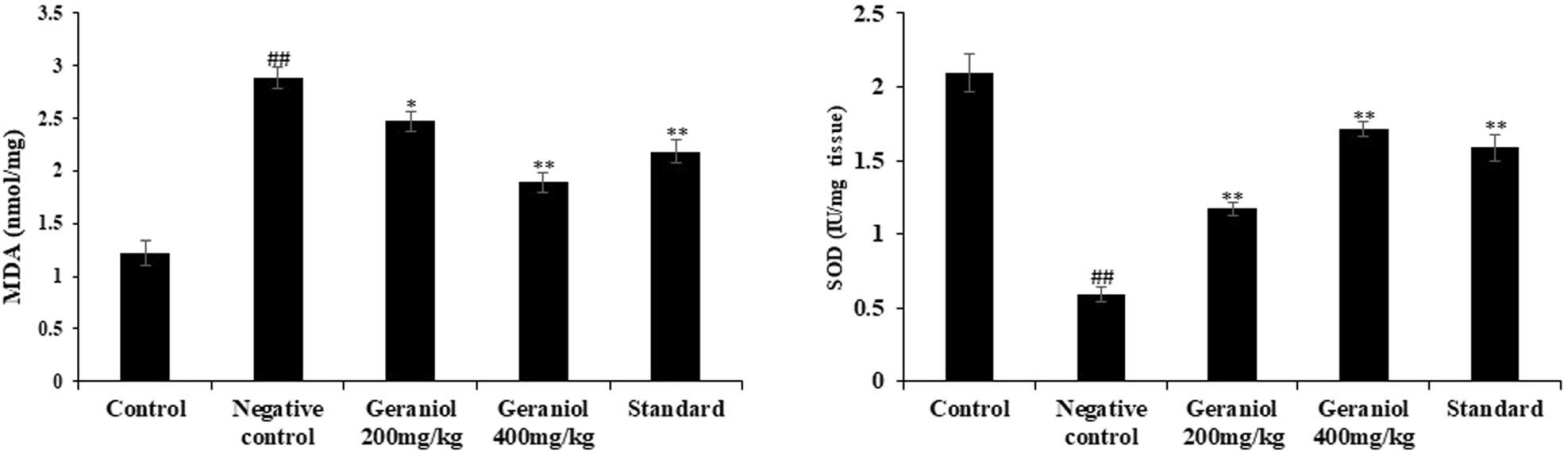
Assessment of geraniol effect on markers of oxidative stress in the liver homogenate of HFD induced obese rats. Mean ± SEM (*n* = 6); ^##^*p* < 0.01 Vs Control; ^**^p < 0.01 Vs Negative control.

Parameters of oxidative stress was assessed in the liver tissue of HFD induced obesity in rats. In negative control group, MDA level was observed to be enhanced (*p* < 0.01) and SOD reduced (*p* < 0.01) in the tissue homogenates than control group. These markers of oxidative stress ameliorated in the liver tissue of geraniol treated HFD induced obesity in rats (Fig. 4).

### Geraniol effect on histopathology of heart, and pancreas tissue

**Fig. 5:**
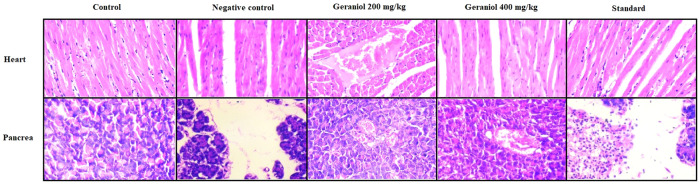
Assessment of geraniol effect on histopathological changes in heart, and pancreas tissue of HFD-induced obese rats. *p < 0.05, **p < 0.01 vs negative control.

Histopathological changes in heart, and pancreas tissue were estimated in the geraniol treated HFD induced obesity in rats (Fig. 5). Heart tissue of control group showed normal morphology, which was observed to be changed as hyaline degenerative changes of cardiac muscle fibre and presence of intramuscular fat in negative control group. Geraniol treated group showed reversal effect on the histopathology of cardiac tissue. Pancreatic tissue appeared to be healthy in control group rats. Pathological changes such as partial evident of degenerated Islets of Langerhans, degeneration in acinar cells as well as increased accumulation of intratubular fat was observed in negative control rats. These pathological changes in the pancreatic tissue attenuated in geraniol treated groups.

### Geraniol effect on WAT and BAT adipose tissue

**Fig. 6:**
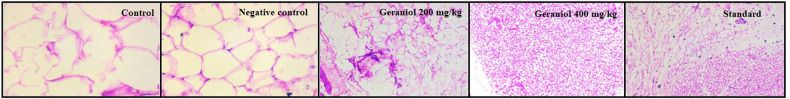
Assessment of geraniol effect on histopathological changes in white and brown adipose tissue of high-fat-diet-induced obese rats. *p < 0.05, **p < 0.01 vs negative control.

Geraniol effect was observed on the white and brown adipose tissue of interscapular region of high fat diet induced obesity in rats as shown in Fig. 6. Histopathology of adipose tissue was observed normal appearance in control group. In negative control group, adipose tissue showed the enlargement of lipid vacuoles which defines higher presence of WAT. However, geraniol treated group showed smaller lipid vacuoles and number of BAT were also found to be enhanced as depicted in histology of adipose tissue (Fig. 6).

### Estimation of geraniol interaction with HMG-CoA reductase (In-silico)

**Fig. 7:**
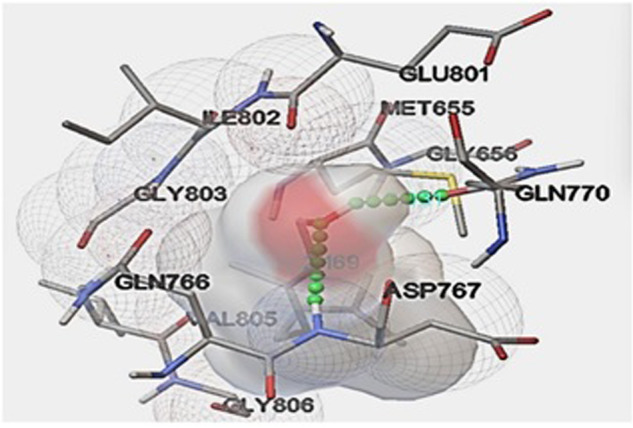
Docking pose of geraniol with the target protein 1HWK. *p < 0.05, **p < 0.01 vs negative control.

Parameters such as number of hydrogen bond forming residues, inhibition constants and binding energies was observed to estimate the ligand-protein interactions are given in Table 2 and docking complexes shown in Fig. 7. Data observed in the study shows maximum binding interaction of geraniol with ASP767 and GLN770 residue of HMG-CoA reductase (1HWK), with binding energy score of ΔG −5.13 Kcal/mole. The bond length involved in this interaction is 2.031 Å, 2.169 Å.

**Table 2:** Interaction of HMG-CoA reductase with geraniol.

Sr. no	Target Protein	Grid centre (xyz-coordinates)	Binding Energy (kcal/Mole)	Hydrogen Bonding residue	Bond Length	VDW Energy	Inhibition Constant
1	HMGCoA Reductase	1HWK	−5.13	GLN 770	2.031	−6.54	174.35
				ASP 767	2.169

## Discussion

Obesity is one the major cause of health issues in the recent era, which is involved in the development of several chronic disorders like diabetes, cardiovascular diseases, and liver disorders [17]. Obesity occurs due to sedentary lifestyle and eating habits, specifically high fat diet and high prevalence appears due to deposition of fat in the adipose tissue. WAT and BAT are major types of adipose tissues and higher amount of WAT is usually observed in individuals with obesity [18]. Whereas BAT is a sign for utilization of stored energy reducing extra calories and the incidences of obesity. Literature reveals that promotion of conversion of BAT into WAT reduces the regulation of energy and develops obesity [19]. Thus, targeting WAT’s conversion into BAT could be used for the management of obesity. Present report evaluates the effect of geraniol on obesity and associated complication in HFD induced obesity in rats.

Obesity is directly related to body weight, HFD reported to promote body weight which also enhanced the WAT deposition [20]. HFD has been reported to be a realistic rodent model of Type II diabetes [21] and data of the present study also supports it. Moreover, literature also suggest that lipid levels get altered in the HFD models and clinically hyperlipidaemia is commonly associated with patients with obesity as HFD promotes the formation of WAT [22]. WAT stores larger volume of lipid (triglyceride), which alters the transportation of GLUT affecting the utilisation of glucose level and reducing sensitivity of tissues for insulin. This in turn leads to enhance the concentration of glucose in the blood causes type II diabetes [23]. Conversion of WAT to BAT causes utilisation of stored energy and burning of calories, reduces body weight and blood glucose. Geraniol was found to decreases the body weight and blood glucose in HFD induced obesity in rats. Data of this investigation suggest that geraniol also ameliorated the altered level of lipid profile and promoted the insulin sensitivity in HFD induced obese rats.

Lipid deposition contributed to the degenerative changes in several organs and also enhanced the weight of these organs including liver, pancreas and heart [24]. Fatty liver is a type of cellular injury to the hepatocytes, that alters the liver function which further promotes the development of several chronic disorders affecting other organs [25]. These fatty changes could be reversed with the balance of lipid profile. Treatment with geraniol reduced the weight of these organs and also ameliorated the altered histopathological changes in these organs. Adipose tissue like WAT majorly available in patients with obesity, is deposited in the different regions of the body. HFD induced rodent model showed the higher quantity of adipose tissue in the different parts of body like sub cutaneous fat, the epididymal, inguinal, mesenteric, triceps, intraperitoneal adipose tissues and data of present report also support these findings [26]. There was decrease in the quantity of adipose tissue in geraniol treated HFD induced obesity in rats. Moreover, histopathology of adipose tissue isolated from interscapular region shows higher number of WAT and lower number of BAT in negative control group compared with control group, which was reversed in geraniol treated HFD induced obesity in rats. WAT represents the deposition of lipid and decrease in insulin sensitivity whereas, BAT is corelated with the heat generation or energy utilisation. This means that geraniol promoted the transition of WAT and BAT and thereby promoted insulin sensitivity.

There are several pathogenic factors involved in the development of disease condition associated with obesity, including oxidative stress and inflammatory pathway. HMG-CoE A reductase activity observed to be reduced in patients with obesity, leads to alteration in the mitochondrial function of adipocytes by the activation of numerous pathogenic pathways including oxidative stress and inflammatory pathways [27]. Oxidative stress alters the cellular functioning by stimulating inflammation, as it leads to mitochondrial dysfunction. This downregulates the UCP1, which is responsible for thermogenesis in BAT and this downregulation reduces thermogenesis and activates the formation of WAT [28]. Treatment with geraniol ameliorates the altered level of oxidative stress and inflammatory mediators. Moreover, data of the in-silico study revealed that geraniol effectively interact with HMG-CoE A reductase and thereby improved the sensitivity of insulin in diabetic rats in this study.

## Conclusion

In conclusion, data of the report revealed that geraniol improved the insulin sensitivity and reduced obesity by promoting the conversion of WAT to BAT in HFD induced obesity in rats. Moreover, geraniol promoted the conversion of WAT to BAT through inflammatory and oxidative pathway regulation by interacting with HMG-CoA reductase.

## Data Availability

The data that support the findings of this study are available from the corresponding author, upon reasonable request.
